# Robotic arm training in neurorehabilitation enhanced by augmented reality – a usability and feasibility study

**DOI:** 10.1186/s12984-023-01225-5

**Published:** 2023-08-12

**Authors:** Alexandra Charlotte de Crignis, Salome-Thamar Ruhnau, Matthias Hösl, Jérémy Lefint, Tamara Amberger, Jürgen Dressnandt, Hans Brunner, Friedemann Müller

**Affiliations:** 1 Schön Klinik Bad Aibling, Bad Aibling, Germany; 2 BEC GmbH, Pfullingen, Germany; 3 Schön Klinik Vogtareuth, Vogtareuth, Germany; 4 Fraunhofer Institute for Manufacturing, Engineering and Automation IPA, Stuttgart, Germany

**Keywords:** Augmented reality, Virtual reality, Active video gaming, Stroke, Neurorehabilitation, Robotic therapy

## Abstract

**Background:**

Robotic therapy and serious gaming support motor learning in neurorehabilitation. Traditional monitor-based gaming outputs cannot adequately represent the third dimension, whereas virtual reality headsets lack the connection to the real world. The use of Augmented Reality (AR) techniques could potentially overcome these issues. The objective of this study was thus to evaluate the usability, feasibility and functionality of a novel arm rehabilitation device for neurorehabilitation *(RobExReha system)* based on a robotic arm *(LBR iiwa, KUKA AG)* and serious gaming using the AR headset *HoloLens* (Microsoft Inc.).

**Methods:**

The RobExReha system was tested with eleven adult inpatients (mean age: 64.4 ± 11.2 years; diagnoses: 8 stroke, 2 spinal cord injury, 1 Guillain-Barré-Syndrome) who had paretic impairments in their upper limb. Five therapists administered and evaluated the system. Data was compared with a Reference Group (eleven inpatients; mean age: 64.3 ± 9.1 years; diagnoses: 10 stroke, 1 spinal cord injury) who trained with commercially available robotic therapy devices (*ArmeoPower* or *ArmeoSpring*, *Hocoma AG*). Patients used standardized questionnaires for evaluating usability and comfort (Quebec User Evaluation of Satisfaction with assistive technology [QUEST]), workload (Raw Task Load Index [RTLX]) and a questionnaire for rating visual perception of the gaming scenario. Therapists used the QUEST, the System Usability Scale and the short version of the User Experience Questionnaire.

**Results:**

Therapy with the RobExReha system was safe and feasible for patients and therapists, with no serious adverse events being reported. Patients and therapists were generally satisfied with usability. The patients’ usability ratings were significantly higher in the Reference Group for two items of the QUEST: reliability and ease of use. Workload (RTLX) ratings did not differ significantly between the groups. Nearly all patients using the RobExReha system perceived the gaming scenario in AR as functioning adequately despite eight patients having impairments in stereoscopic vision. The therapists valued the system’s approach as interesting and inventive.

**Conclusions:**

We demonstrated the clinical feasibility of combining a novel robotic upper limb robot with an AR-serious game in a neurorehabilitation setting. To ensure high usability in future applications, a reliable and easy-to-use system that can be used for task-oriented training should be implemented.

**Trial registration:**

Ethical approval was obtained and the trial was registered at the German Clinical Trials Register (DRKS00022136).

## Background

Neurological rehabilitation is crucial to public health, as a substantial part of the population is affected by neurological diseases throughout their life. Stroke, for example, has a one-year-prevalence of about 1.6% in the German adult population, increasing with age to over 6% in people over the age of 75 [1]. Considering the demographic change, the number of persons suffering from lasting disabilities after stroke are projected to increase [2, 3].

After damage to the central nervous system, neural plasticity enables the brain to adapt in response to motor learning [4]. To achieve this, it is recommended to perform a highly intensive, repetitive and task-specific rehabilitation [5]. However, this requires extensive time and personnel investments. Moreover, the patient’s attention and motivation are crucial for acquiring high dosages of training [6]. Both robot-assisted therapeutic devices [7] as well as serious gaming approaches [8] address those challenges. They are already combined in many commercially available robotic devices for the upper limb (e.g., ArmeoPower (Hocoma AG, CH) or AMADEO (Tyromotion GmbH, AT)) supplementing neurorehabilitation [9, 10]. These systems commonly use a standard monitor for displaying the gaming environment, which may lack an intuitive representation of the third dimension (depth) and thus the possibility to practice relevant everyday life movements and tasks. Head-mounted displays as the gaming environment manage to represent a virtual, realistic third dimension [11]. However, using fully-immersive virtual reality glasses may induce motion sickness, as there is a complete loss of reference to the real world. The use of Augmented Reality (AR) head-mounted displays, such as the HoloLens (Microsoft, US), seem to overcome this issue [12].

AR generally has beneficial effects for the learning of spatial tasks [13]. First attempts have introduced AR technology in neurorehabilitation demonstrating overall good feasibility and user experience [14]. To our knowledge, the HoloLens with a head-mounted AR-display has only been used with neurological patients in a few exploratory trials; for example, Rohrbach et al. (2019, 2021) used the HoloLens to improve the pantomime performance of patients with apraxia and to support ADL tasks in patients with dementia. Further applications were in patient education before surgery of epilepsy patients [15], in gait training in patients after stroke [16] or with Parkinson’s Disease [17] or during the evaluation of vision in patients after stroke [18]. Concerns have been raised that elderly and/or persons with cognitive, sensory and visual impairments (which might occur due to stroke) face difficulties in perceiving the three-dimensionality of an AR environment [18]. Previous research has shown that, in patients after stroke using the HoloLens, impairments in stereovision and consequently depth and distance judgement within the AR environment were present [18].

Therefore, in patients with upper limb disabilities due to stoke, our approach was to combine AR with a robotic device to administer a serious gaming therapy. We were also particularly interested if it was possible to create a gaming situation with an easily perceptible depth dimension.

The objective of this pilot proof-of-concept study was to evaluate the device’s safety, clinical feasibility, usability, and potential benefits, as recommended by Maciejasz et al. [19].

## Methods

### User

This study had three groups of users: eleven patients (“RobExReha-Patients”) (aged 64.4 ± 11.2 years, range 47–85) and five therapists (“RobExReha-Therapists”) (aged 38.2 ± 16.0 years, range 23–57) who evaluated the RobExReha device and an additional eleven age-matched patients (“Reference Group”) (aged 64.3 ± 9.1 years, range 49–79) were allocated to the reference group (see Table 1). The RobExReha-Patients group received, and the RobExReha-Therapists group administered training sessions using the RobExReha device; the Reference Group trained with state-of-the-art commercially available and established devices (ArmeoSpring and ArmeoPower, both Hocoma AG, CH). Both devices are typically used for arm and hand therapy in neurological or orthopedic rehabilitation and have proven to be effective [20–22]. Moreover, as established devices, they provide a good usability [23] and allow therapists to supervise more than one patient at a time [24]. They both combine a mechanical support of the upper extremity (either passive by a spring-loaded system (ArmeoSpring) or actuated (ArmeoPower)) with a variety of serious games presented on a standard monitor. These devices will not be further described hereinafter as they are widely distributed and known.

Inclusion criteria for patients were a subacute or chronic paresis of the upper limb due to neurological disorders, preserved language comprehension, ability to communicate, orientation in space and time, ability to sit upright for at least 45 min, and the completion of at least four training sessions with the respective therapy device (RobExReha or Armeo). Further device-related inclusion criteria for the RobExReha-Patients group were ability to reach the clip-in-position of the robotic device at 30° abduction of the shoulder, impairment on the right side, and normal or appropriately corrected vision.

We excluded patients with craniotomy, instable fractures, fixated contractions, active implants, epilepsy and severe instabilities, severe spasticity (Modified Ashworth Scale > 3, [25]) or open skin defects of the affected upper limb. Further, patients were screened by the treating neurologist for severe neuropsychological problems such as apraxia, severe neglect, severe aphasia or dementia.

Stereoscopic vision was evaluated using the Titmus-Test (Stereo Optical Co., Chicago, IL) but was not a limiting factor for study inclusion. The mean stereoscopic vision of the RobExReha-Patients group was 405 ± 983 arc sec (median: 100 arc sec; range 40–3352 arc sec). With defining the cut-off score for unimpaired stereoscopic vision at 60 arc sec [18], seven patients showed impairments in stereoscopic vision and four patients were able to see stereoscopically well. All except one patient were able to see better or equivalent to 400 arc seconds.

The five therapists administering the therapy had a working experience of 9.8 ± 8.2 years in neurorehabilitation (min-max: 1–23 y.). They were either physiotherapists (n = 2), a sport therapist (n = 1), a health scientist (n = 1) or a specially trained staff member for robotic upper limb therapy without specific health care profession (n = 1). Two of the therapists were highly trained using other robotic devices daily, and three of them had not regularly used robotic therapy. The technical affinity questionnaire (TA-EG) [26] was administered to describe their technical affinity. This questionnaire evaluates the positive and negative attitudes as well as competence and enthusiasm towards technology. The items are answered on a five-point Likert-Scale. The result is the mean value of the items with 1 indicating a low, and 5 indicating a high technical affinity. The therapists had an overall medium to high technical affinity, with an average of 3.4 (min: 3.1 max: 4.1) points (out of 5) in the TA-EG.

**Table 1:** Characteristics of participants

	RobExReha-Patients	RobExReha-Therapists	Reference Group
Sample size	11	5	11
Age in years *M ± SD (range)*	64.4 ± 11.2 (47–85)	38.2 ± 16.0 (23–57)	64.3 ± 9.1 (49–79)
Gender *(male/female)*	9/2	1/4	9/2
Diagnosis	Stroke (n = 8) Spinal cord injury (n = 2) Guillain-Barré-Syndrome (n = 1)	-	Stroke (n = 10) Spinal cord injury (n = 1)
Time since paresis in days *M ± SD (range)*	347 ± 611 (35-2151)	-	257 ± 403 (32-1452)
Affected side *(left/right/both)*	-/7/3	-	7/3/1

M: mean, SD: standard deviation

All participants gave informed written consent. Ethics approval was obtained (Ethikkommission der Bayerischen Landesärztekammer) and the study was registered in the German Clinical Trials Register (DRKS00022136).

### Technology

**Fig. 1:**
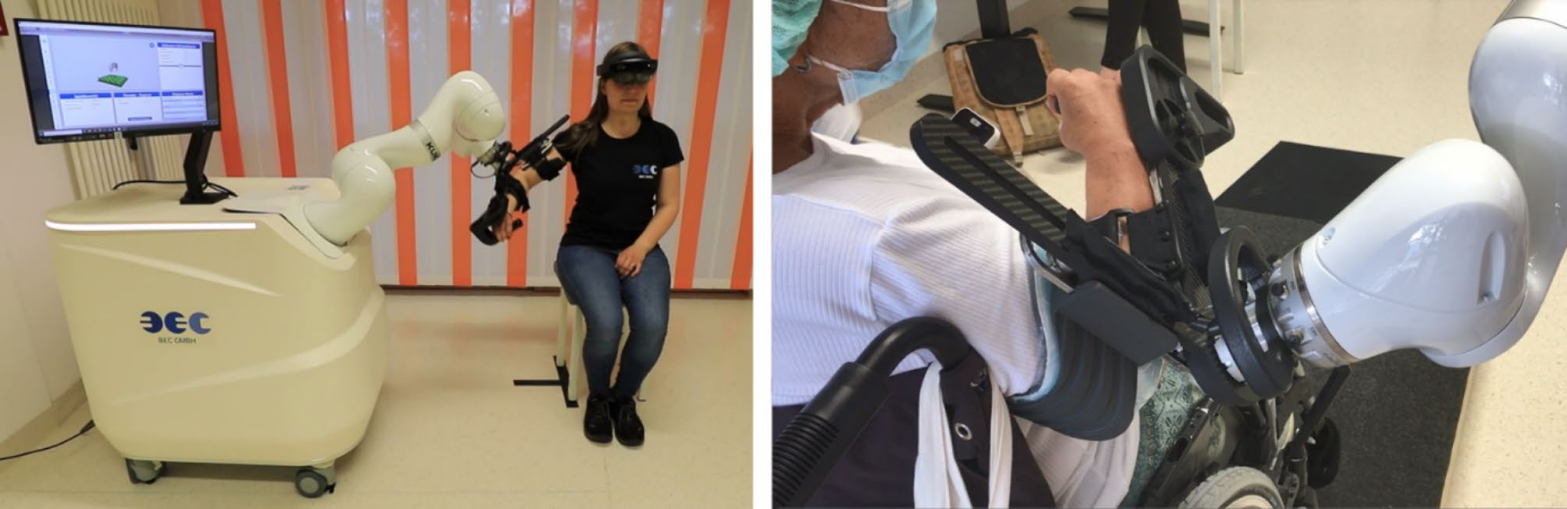
Left: subject using the RobExReha system with the HoloLens for the gaming application. Right: arm-robot interface used by a patient during the therapy with the RobExReha system

**Fig. 2:**
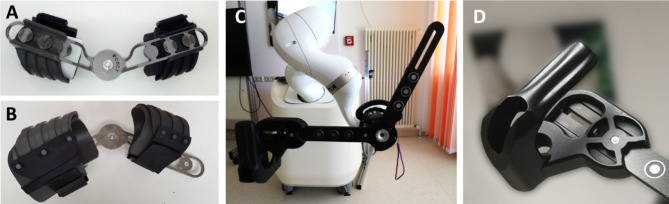
Arm-robot interface of the RobExReha system: Picture **A** and **B** show the elbow brace, picture **C** shows the counterpart on the robotic flange, which consisted of two carbon fibre plates with three electromagnets attached on each. The two plates were connected via a joint at the robot flange. The plate supporting the upper arm was rotatable, while the second plate for the forearm was fixed on the flange and could therefore be controlled by the robotic movement. Picture **D** shows the hand module

The RobExReha robotic system should meet the demand to provide (adaptive) support to the patient, while ensuring a high level of safety. Therefore, the commercially available LBR iiwa robotic arm (KUKA AG, DE) was chosen, which is designed for safe human-robot interactions. The robotic arm was mounted on a cart in a 45° angle and interfaced with the patients’ right arm at the robot flange (see Fig. 1). The robotic setup was further interfaced with a custom-made Unity application (Unity Technologies, US) for the HoloLens (Microsoft Inc., US) that enabled patients to perform active serious gaming with robotic support for the impaired arm (see Fig. 2). The Microsoft HoloLens (Microsoft Corp., US) was chosen as the AR device, as it had already been implemented in various contexts (e.g., surgical aids, medical education, industrial engineering) [27], including in stroke [28]. The different components were linked within a separate network via ethernet cables and the HoloLens was wirelessly connected. Robot information including position of flange and axes as well as active forces for data analyses were saved with a sampling rate of 25 Hz.

#### Arm-robot interface

To connect the patient’s arm to the robot flange, a mechanical human-machine interface comprising a brace on the upper and lower arm and a counterpart on the robotic flange was developed. The brace consisted of two 3D printed shells (Fig. 2), which were available in two different sizes. The upper and lower arm shells were connected via a ferromagnetic steel plate, that featured a joint at the height of the elbow (see Fig. 2). The brace was donned on the patient’s arm by a therapist and fixed with Velcro straps.

Subsequently, the steel plate of the brace (Fig. 2A/B) could be attached magnetically to the carbon plates fixed at the flange (Fig. 2C). It was thereby ensured by a bolt and stop to prevent the angle of the elbow from exceeding 180°. The electromagnets for the connection of the brace had a nominal force of about 210 N. This was strong enough to hold the brace with the arm in position but weak enough to release it quickly by a therapist in case of emergency.

A hand module with a grip hold was attached to the carbon plate that guided the lower arm (see Fig. 2D). The position of the hand module could be easily adjusted to the length of the lower arm with a screwed connection.

#### Calibration procedure

To calibrate the position of the robot, the shoulder height and upper arm length were manually measured using a measuring tape in the beginning of the first session and entered in the software. At the beginning of each session, the robot moved the flange to the respective clip-in position at 30° shoulder abduction. Once the brace was clipped to the arm, the arm was weighed by the robot to enable the calibration of robotic support. Afterwards, the patient’s specific range of motion (ROM) was evaluated, which consisted of two assessments: the active ROM, in which the patient could actively move the impaired arm, and the passive ROM without pain as guided and determined by the therapist. The passive ROM typically exceeded the active ROM in patients. This differentiation enabled the implementation of two different robotic support modes: (A) The support level was set to a constant level for both ranges, or (B) the support level within the active ROM was set to a constant level while the support of the movement beyond the active ROM increased dynamically until the patient achieved the desired movement. The robot enabled six support levels that were adjustable with increments of 20%, from 0 to 100%.

#### Safety features

To ensure the patients’ safety, the robot’s movements were restricted to the respective patient’s passive ROM. An additional safety feature was an automated stop in case the active sum force of all axes exceeded 30 Nm. Moreover, a safety stop button for the supervising therapist was implemented.

To give feedback about compensatory movements of the trunk or shoulder girdle, an alert was implemented to notify both therapists and patients if the patient’s shoulder position (i.e., the acromion) left the anticipated position. In case this position deviated more than a predefined threshold from the initial shoulder position, a visual alert was displayed in the gaming environment. Initially, the threshold of this shoulder compensation alert was set to a range of 5 cm around the ideal shoulder position. However, the alert tended to be displayed too early and too frequent and thus interrupted the gaming experience. After increasing the threshold to 7.5 cm, the alert only appeared in cases where a relevant compensatory movement was visually evident for the supervising therapist. The patients reacted adequately by actively correcting the position of their shoulder.

#### Gaming scenario

**Fig. 3:**
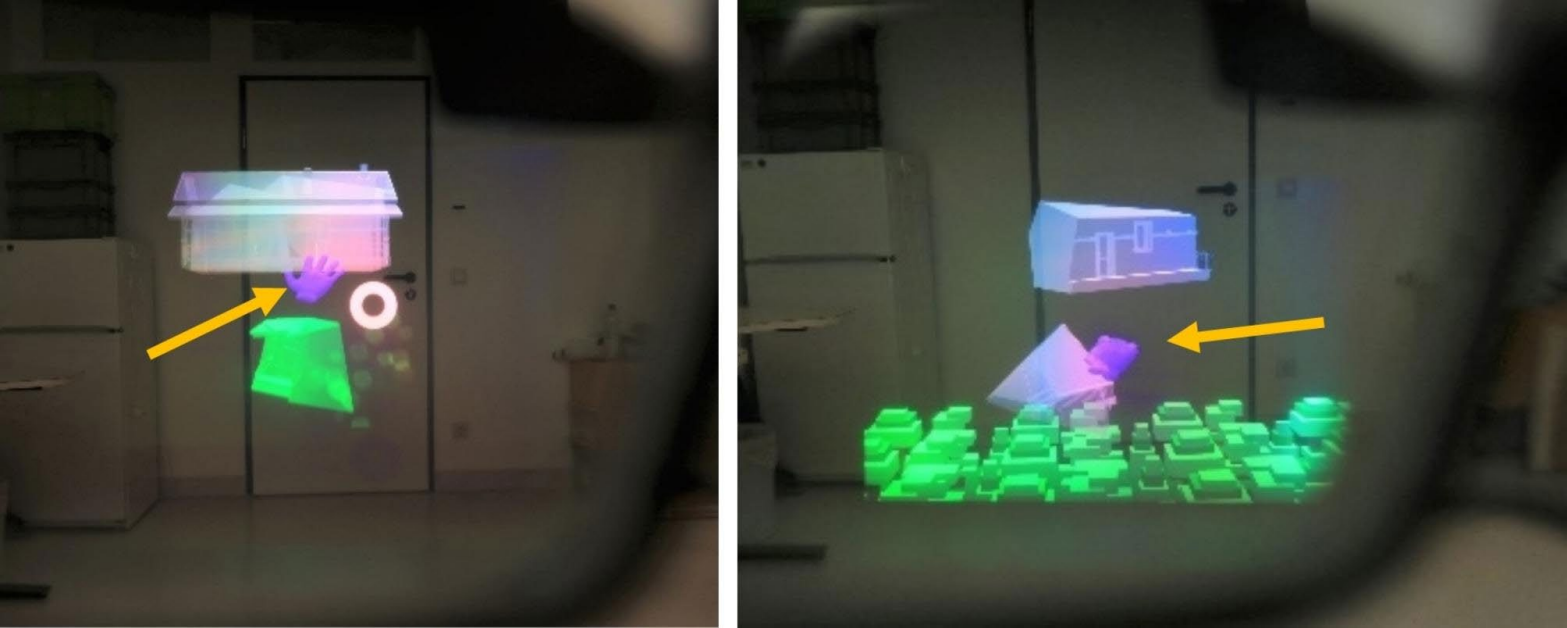
View through the HoloLens with the gaming scenario: a blue hand (yellow arrows) could select puzzles pieces. Once a piece was selected, the hand closed (see right figure) and the piece could be moved to the building

The training setup included an AR-game for HoloLens. The training game was based on a 3D puzzle (see Fig. 3): A building (e.g., tower, house) and a blue hand appeared within the field of view of the patient. Subsequently, the building fell into pieces, with its contours remaining. The pieces were spread in front of the building within the patient’s specific active ROM. The designated target positions for the rebuilding process of the building were within the range of the patient’s passive ROM. The blue hand served as controller and it was moved by the position of the real hand. Once the virtual hand approached a puzzle piece, the virtual fingers grabbed the piece. Next, the respective target position of the grabbed piece was indicated and the piece could be moved to the target position, automatically being released by the hand.

The difficulty of the game could be adjusted via the distance and size of the building with respect to the hand position, which led to a smaller/greater required travel distance of the patient’s hand to reach the designated spot. This, along with the robotic support level, could be also adjusted while gaming.

### Study protocol

**Fig. 4:**
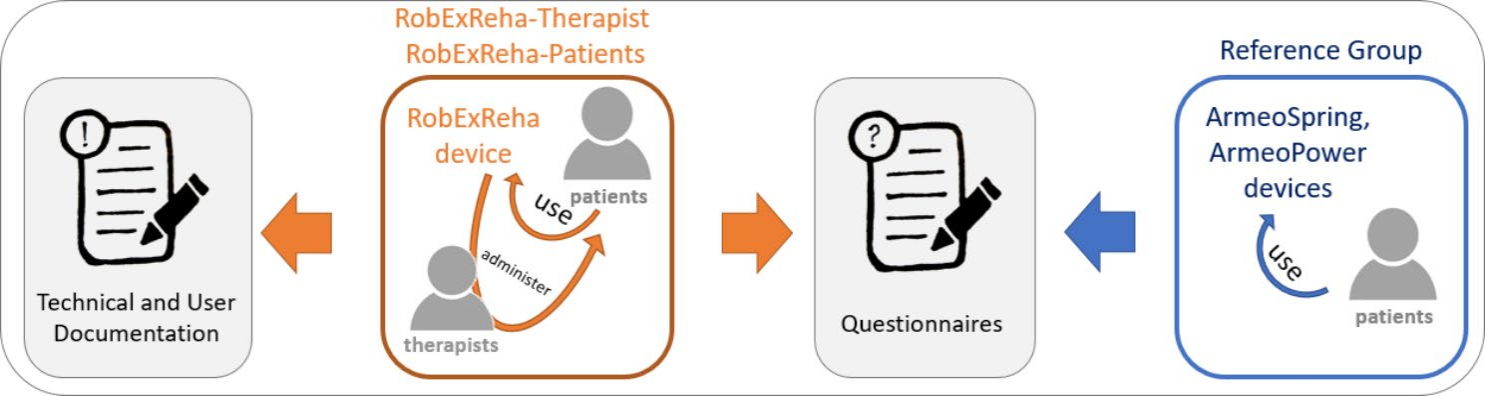
Schematic of the structure of this study: the RobExReha-Patients trained 4–5 times with the RobExReha device, while technical and user incidents were reported. After completion of at least four training sessions, the questionnaire evaluation was conducted. The Reference Group only participated in the questionnaire survey and reported their perception of the conventional robotic gaming therapy

To evaluate the usability and feasibility, the groups used the devices (either directly as a patient or administering the therapy as therapist). After at least four sessions, their experience was evaluated using questionnaires (see Table 2). Additionally, during the training with the RobExReha device, all technical difficulties, user errors or safety aspects were documented (Fig. 4). Ten out of eleven patients of the RobExReha-Patients group additionally completed an extra (fifth) therapy session which was recorded using a video protocol to investigate the donning and set-up time of the RobExReha device.

#### User activity

##### **RobExReha-patients group**

**Fig. 5:**
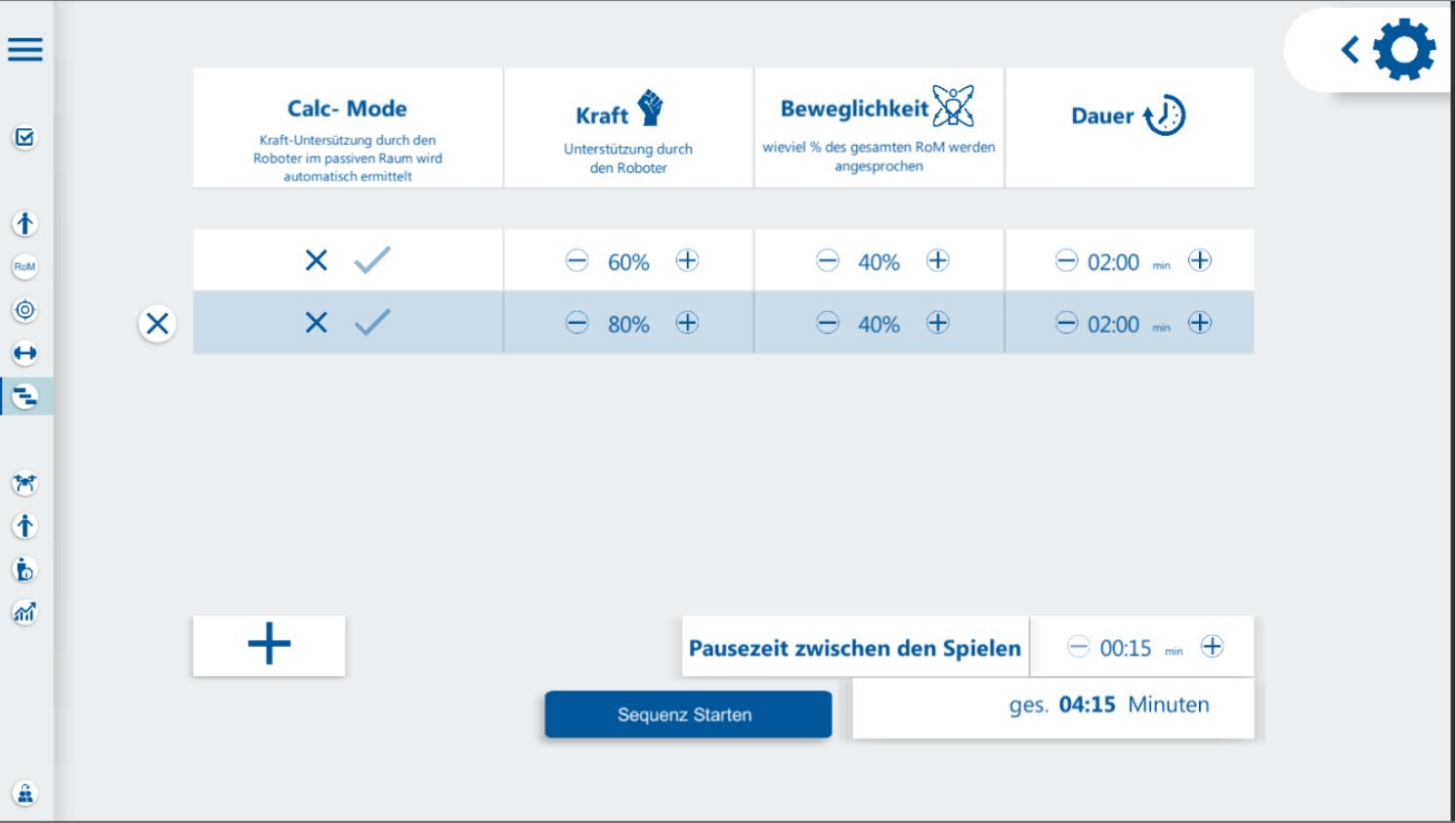
User Interface of the RobExReha system for the therapist. In this screen, the therapy session could be planned and adapted: Separate gaming sessions could be added and adjusted in length (german description: “Dauer”), as well as the rest length between the sessions (“Pausezeit zwischen den Spielen”). Additionally, the requirements in terms of range of motion (“Beweglichkeit”) and the support by the robot (“Kraft”) could be adjusted via this interface by the therapist. Under the “Calc-Mode” the paradigm of robotic support (adaptive/non-adaptive) could be chosen;

Patients in the RobExReha-Patients group received training with the RobExReha device. A session consisted of the donning and setup of the device, subsequent gaming therapy, termination of the session and doffing of the device. Amount and mode of robotic support as well as the length of the gaming sessions were adjusted by the therapist according to the patient’s needs. Figure 5 shows the User Interface to configure these parameters.

##### **RobExReha-therapists group**

The therapists administered the RobExReha device in at least five sessions to a patient before answering the questionnaire. They were responsible for donning, doffing, and configuring the training session as well as supervising the patient.

##### **Reference group**

The patients in the Reference Group received rehabilitation training with the commercially available ArmeoPower or ArmeoSpring (Hocoma AG, Switzerland) as part of their inpatient schedule. Six patients in the Reference Group trained with the ArmeoPower device and five with the ArmeoSpring device, with a mean of 15 (± 25, min: 4, max: 90) completed therapy sessions. They received no further intervention regarding this study. This data acquisition was done during the developmental stage of the RobExReha device and thus before the data acquisition of the RobExReha device.

#### Questionnaires

**Table 2:** Overview over the standardized questionnaires used for the usability evaluation

Questionnaire	RobExReha -Patients	RobExReha-Therapists	Reference Group
Device subscale of QUEST	X	X	X
Raw Task Load Index	X	-	X
pAR	X	-	-
SUS	-	X	-
UEQ-short	-	X	-

QUEST: Quebec User Evaluation of Satisfaction with assistive technology, pAR: presence in augmented reality questionnaire, SUS: System Usability Scale, UEQ-short: short version of the User Experience Questionnaire

##### **Patients’ questionnaire**

Both patient groups filled out the Quebec User Evaluation of Satisfaction with assistive Technology (QUEST) [29] and the Raw Task load index (RTLX) [30]. The QUEST was designed to measure the level of satisfaction attributable to assistive technologies. We used the device subscale score (8 items), which was scored in terms of perceived satisfaction from 0 to 5 (5 ~ highly satisfied) and had been previously applied to measure satisfaction with rehabilitation and assistive robotic devices [31]. We used the QUEST without individual weighting of results and item four was changed to “reliability and safety” in deviation from the original scale. The item “how satisfied are you with the durability of the device?” was excluded, as no meaningful evaluation after the intervention period of four to five sessions was possible. The RTLX evaluates the workload using six subjective subscales: mental, physical and temporal demand, performance, effort and frustration. Each item is rated within a 100-points range. The mean value is calculated to receive the overall task load index. We used the raw version without individual weighting of these subscales. A value of 100 suggests very high task demands or very high failure in terms of performance [32].

As the RobExReha-Patients group used the HoloLens for the gaming environment, they additionally filled out the “presence in augmented reality” (pAR) questionnaire (adapted from [33]). The pAR questionnaire evaluates the patients’ impression of the output of the HoloLens. The questionnaire was adapted by turning questions into statements that could be answered with a 5-point Likert scale (5 ~ totally agree).

##### **Therapists’ questionnaire**

The therapists completed a questionnaire consisting of the same adapted device subscale of the QUEST as the patients, the system usability scale (SUS) [34] and the short version of the User Experience Questionnaire (UEQ-short) [35] to express their perception of the device’s usability.

### Statistical methods

The results were evaluated descriptively and the results of QUEST and RTLX from the RobExReha-Patients and the Reference Group were compared using Mann-Whitney U-test. Due to the exploratory character of the study, no correction for multiple comparisons was done. Analyses were done in Matlab (The MathWorks Inc., US) and SPSS (IBM, US). The alpha level was set to 0.05 for all analyses. Data is presented as either the mean (± standard deviation) or the median (min-max).

## Results

### Safety

No serious adverse events occurred and none of the patients mentioned feeling unsafe at any time. However, one study-related non-serious adverse event was registered: temporary shoulder pain, which vanished within an hour after the therapeutic intervention. This patient had an unstable sitting posture in the wheelchair, which likely contributed to the shoulder pain.

Three of 54 therapy sessions had to be terminated earlier due to technical problems and four due to patient reasons (lack of attention, discomfort in the wheelchair or onset of shoulder pain).

### Usability

#### Feasibility and technical aspects

Concerning the robotic set up, the positioning of the LBR arm on the cart (Fig. 2) was found to be unfavorable as it sometimes led to restrictions of certain parts of the abduction-elevation movements. Patients perceived this, as if the robot sometimes was “working against them”. Further, they mentioned that the system was not always operating smoothly (e.g., due to connection errors, software errors or safety stops of the LBR robot), which led them to rate the “reliability and safety” of the RobExReha system as the least satisfying component in the QUEST questionnaire. Regarding the HoloLens, three patients perceived the glasses as slightly unhandy, heavy or ill-fitting, which led to lower scores in the questionnaires (e.g., QUEST). In another five instances, the glasses slipped onto the nose and required readjustment by the therapist.

For three patients, the arm weighing process did not function as intended. As a result, the default weight of 0.01 kg was shown by the robot and the weight needed to be estimated and corrected manually. These patients suffered pronounced spasticity in the elbow flexors.

#### Video protocol – donning and doffing times

Starting and preparing the RobExReha system included the activation of the system and HoloLens, the connection of the HoloLens to the robot, donning the elbow brace onto the patient’s arm and connecting it to the robot flange, weighing of the arm as well as preparation and donning of the HoloLens. This collectively took a median of 293 s (range 173–452 s). The two most time-consuming steps of the starting procedure were donning the HoloLens, which took a median of 49 s (10–151 s), and donning of the elbow brace, which took a median of 47 s (40–100 s).

Termination and doffing of the RobExReha system included a pain assessment, disconnecting the brace from the robot and doffing of the brace and HoloLens. In total, this took a median of 67.5 s (29–114 s). Doffing the HoloLens took a median of 17.5 s (13–37 s). Disconnecting the brace from the robot was perceived as easy, which was reflected by the median disconnection time of 12 s (6–28 s).

#### Questionnaires

##### **Patients’ perspective - usability**

**Fig. 6:**
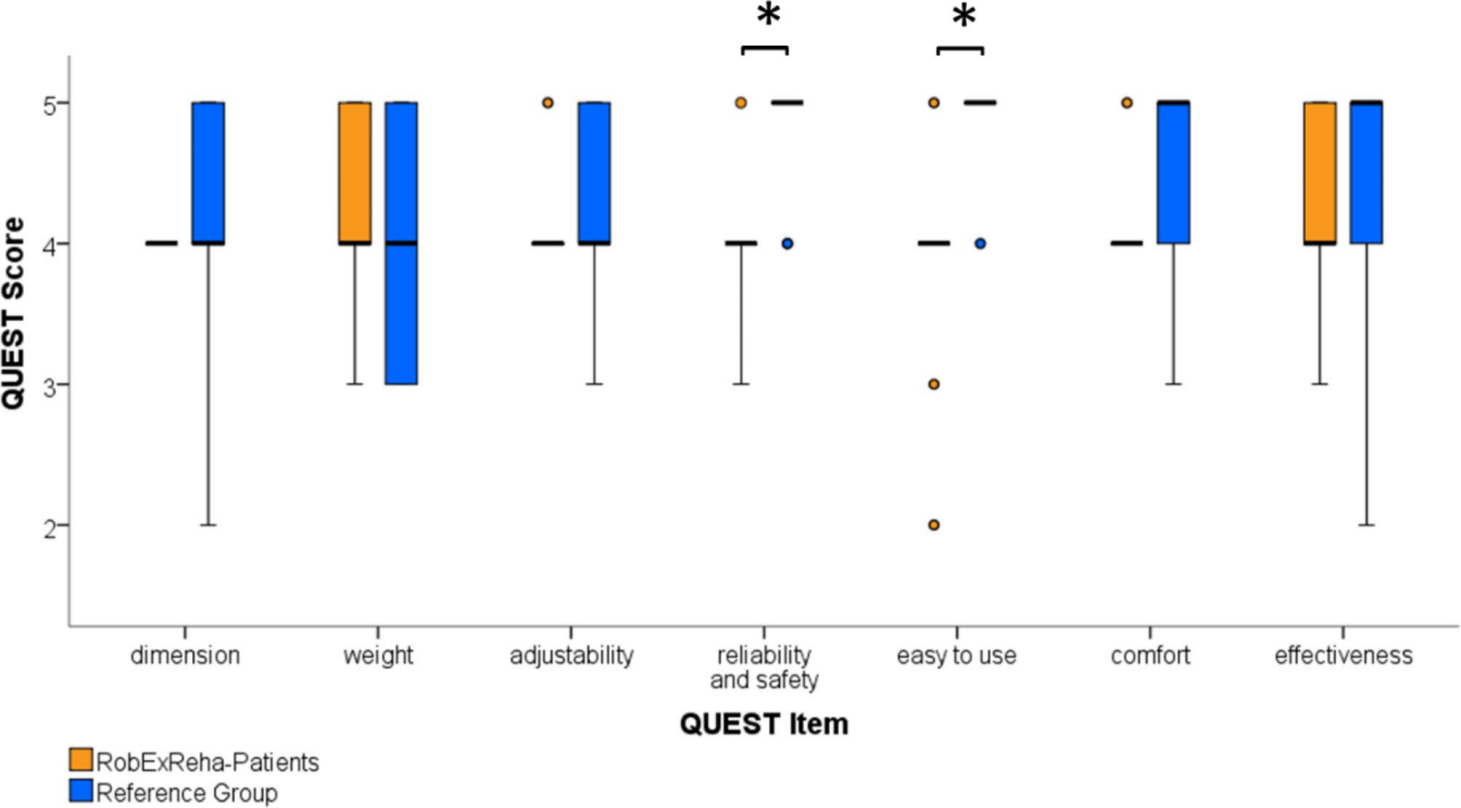
Results of the Quebec User Evaluation of Satisfaction with assistive technology (QUEST) patients’ questionnaire: Scores can range from 1 (not satisfied at all) to 5 (highly satisfied). Asterisks indicate significant differences. Circles indicate outliers

The results of the QUEST (see Fig. 6) show that patients were generally satisfied with the evaluated aspects of the robotic arm therapy with median values being 4 or 5 (~ satisfied/very satisfied) for all items in both groups. The overall results from the RobExReha-Patients group (Mean 3.9 ± 0.4) differed significantly (p = 0.02) from the ratings by the Reference Group (Mean 4.4 ± 0.4). Item-wise, there were significant differences for the items “reliability and safety” and “easy to use” (both p < 0.001). Regarding the other items, no significant differences were found (p > 0.07).

The RobExReha-Patients found the arm orthosis, grip module and HoloLens AR-glasses to have limitations: Four patients mentioned that the orthosis (especially the part for the upper arm) could be more comfortable. Four patients found the grip module to be uncomfortable or not be adaptable enough (in length and/or in wrist motion). Two patients mentioned the HoloLens being uncomfortable or heavy.

##### **Patients’ perspective - task load**

**Fig. 7:**
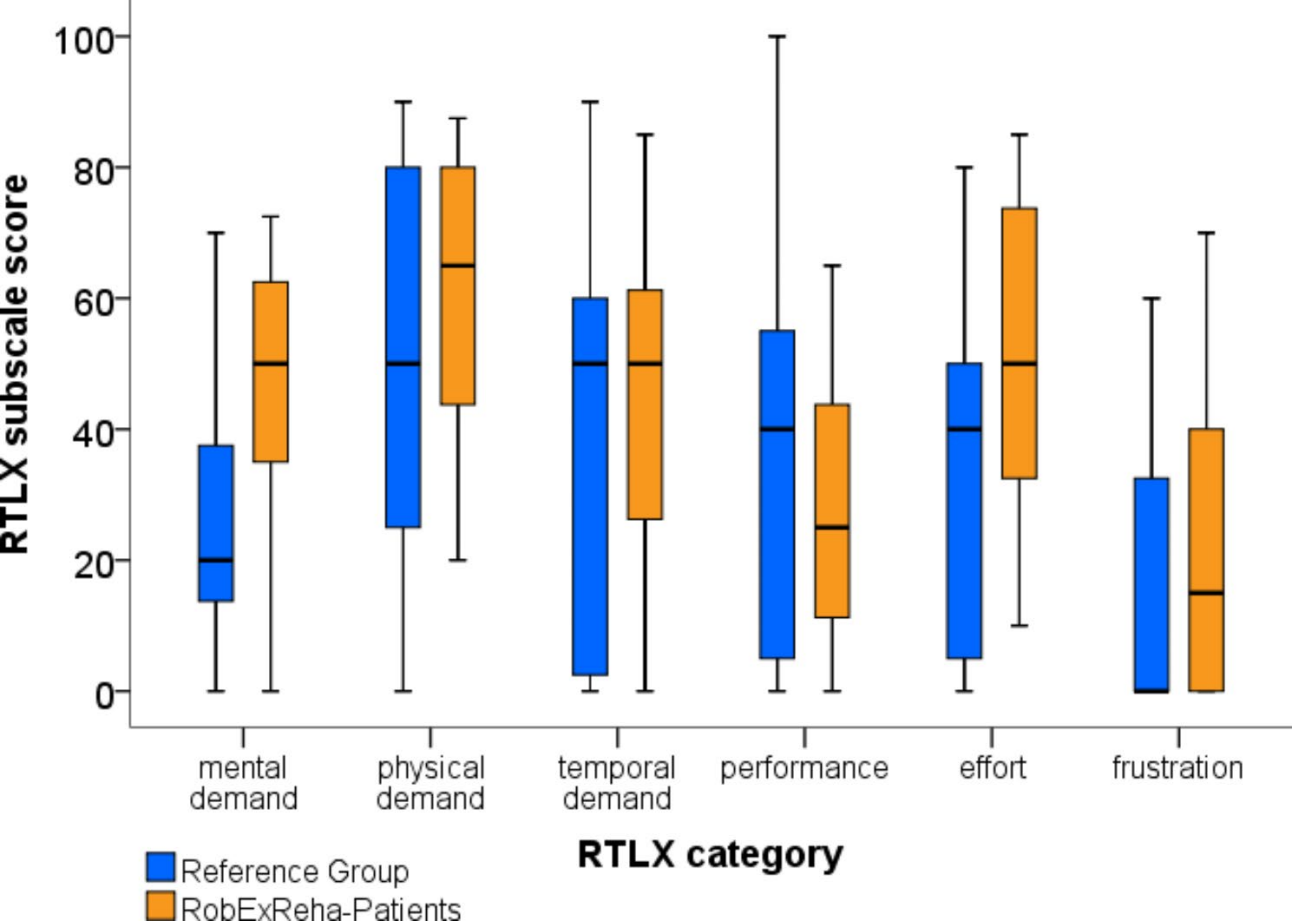
Results of the -Raw Task Load Index (RTLX) subscale scores displayed as boxplots

The results of the RTLX questionnaire are displayed in Fig. 7. Mann-Whitney-U-Test revealed no significant differences between the groups for each RTLX category (p ≥ 0.06). The overall mean RTLX score was 41.7 (± 12.6)for the RobExReha-Patients group and 34.1 (± 15.8) for the Reference Group (p = 0.22).

##### **Patients’ perspective − 3D perception**

**Fig. 8:**
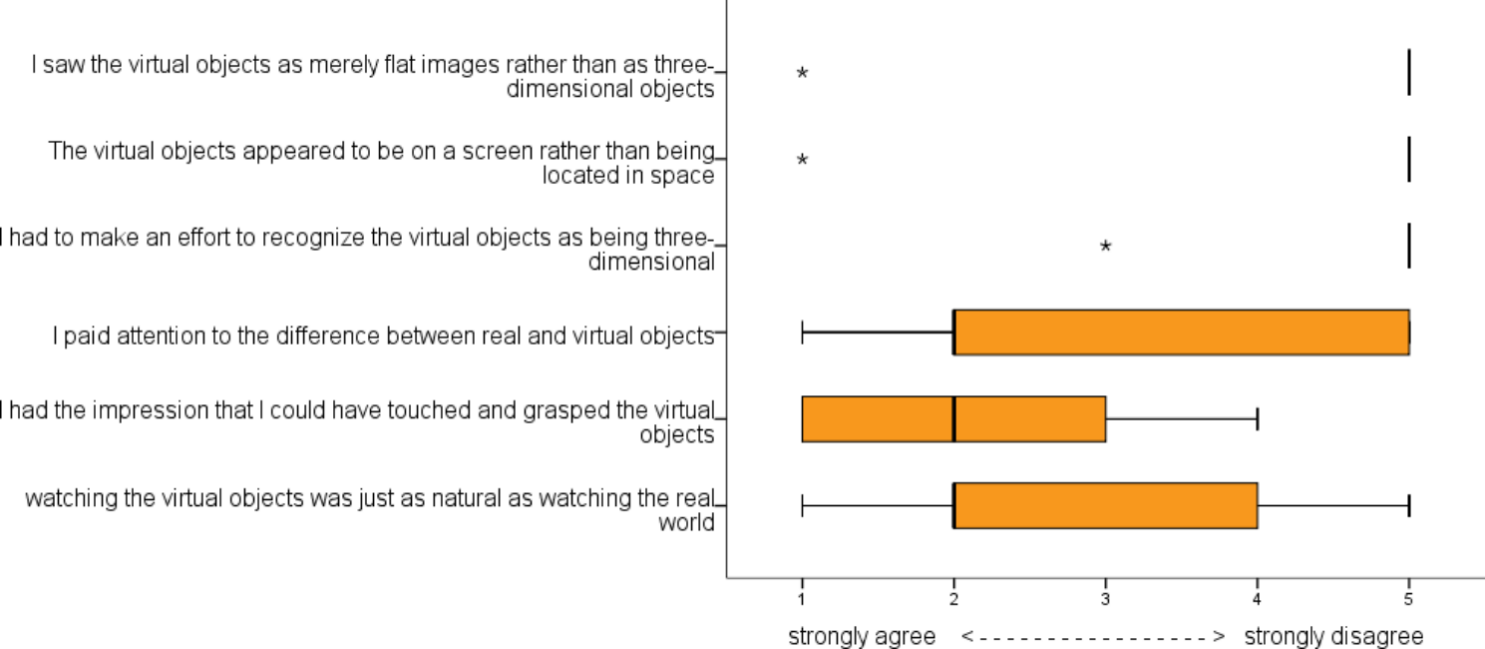
Results of the presence in Augmented Reality (pAR) questionnaire

The results from the pAR questionnaire are shown in Fig. 8. All except one patient perceived the objects as three-dimensional and situated in space. This patient, however, remarked that although he saw the virtual objects as merely flat, progressively he adapted and learned to perceive them three-dimensionally and located in space. This patient had only minor impairments in stereoscopic vision (ability to see 80 arc sec in the Titmus test). Regarding the other statements, the results were less concurring. However, considering the median values, most patients (1) paid attention to the difference between real and virtual objects, (2) had the impression that they could have touched and grasped the virtual objects and (3) felt that watching them was just as natural as watching the real world.

Further remarks from the patients regarding the visualization of the game through the HoloLens concerned the limited field of view. This led to difficulty in understanding the game (two patients) and the process of applying and adjusting the glasses, which one patient perceived as taking too much time.

##### **Therapists’ perspective**

**Fig. 9:**
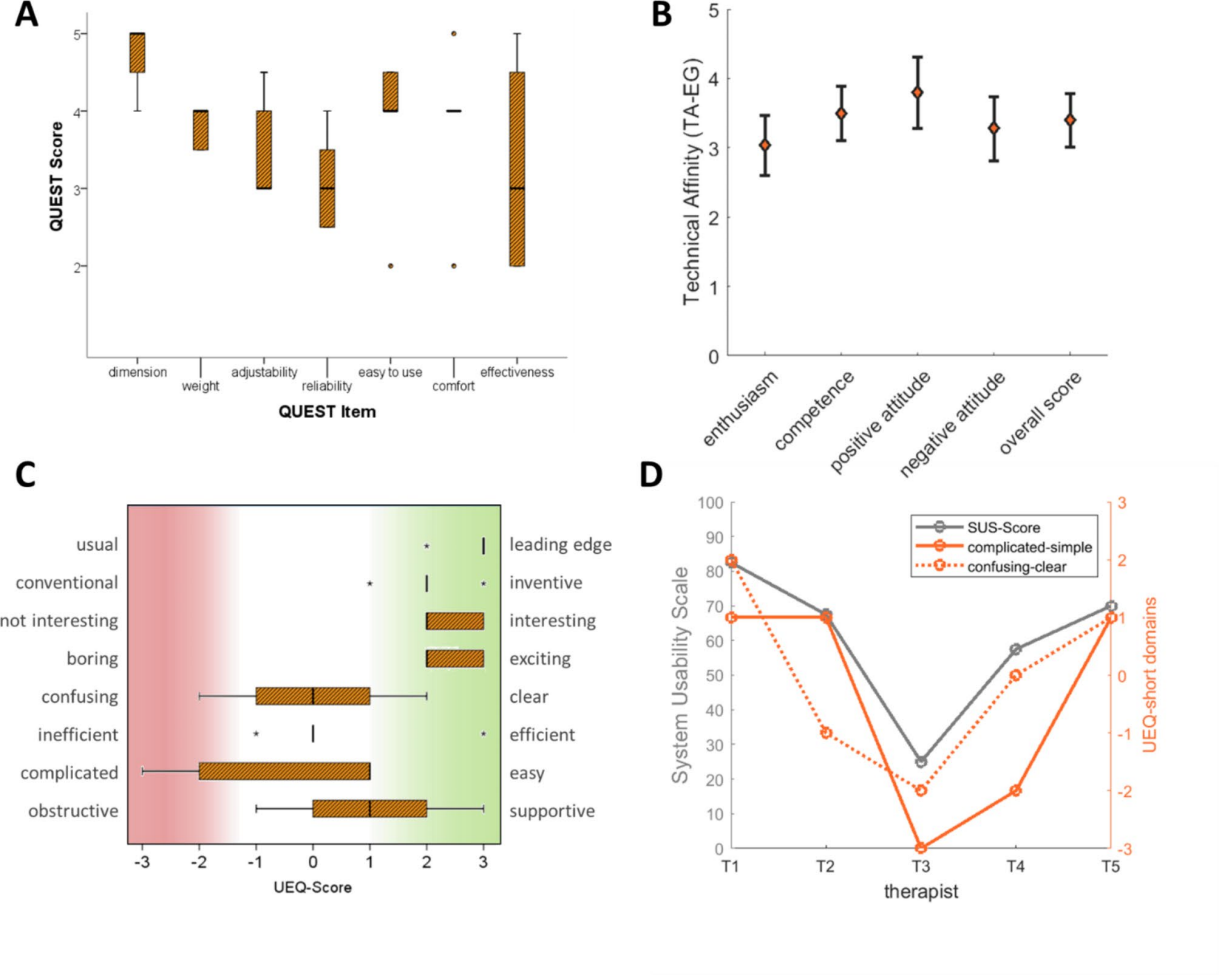
Results of the evaluation by the RobExReha-Therapists Group: **(A)** Results from the Quebec User Evaluation of Satisfaction with assistive Technology (QUEST) Questionnaire, please note, that the therapists answered this twice (for the robotic part and the HoloLens gaming part of the device). The mean of these two are reported (QUEST-Score from 0 to 5; 5 ≈ highly satisfied) **(B)** Results of the technical affinity Questionnaire (TA-EG) (TA-EG-Score from 1 to 5; 5 ≈ high technical affinity) **(C)** Results of the User Experience Questionnaire-short (UEQ-short) and **(D)** the individual results from the System Usability Scale (SUS-Score: 100 ≈ very good usability) and selected items of the UEQ-short: (-3 ≈ negative rating, 3 ≈ positive rating) (right). T = Therapist.

The therapists that administered the RobExReha system rated the usability overall slightly lower in the QUEST (mean: 3,7 ± 0.7, range: 2.8–4.6) than the patient groups. The items that were ranked lowest were “reliability and safety” (3.1 ± 0.7), “effectiveness” (3.3 ± 1.5) and “adjustability” (3.5 ± 0.7). The QUEST item ranked highest by the therapists was the “dimension” (mean: 4.8 ± 0.5). The overall mean usability as measured with the SUS was 60.5 (SD 21.8 range 25-82.5). The UEQ-short (Fig. 9C) revealed that the device was perceived as “*exiting*”, “*interesting*”, “*inventive*” and “*leading edge*” by all therapists. Regarding the clearness and efficiency, there was an indecisive rating with a median at 0 points. Some therapists experienced the RobExReha system as more complicated, however, the median tended slightly towards easy. Further, in the therapists there was a tendency to rate the system as supportive rather than obstructive. No correlation between the technical affinity and the SUS or UEQ scores could be found. However, the two subscales “complicated – simple” and “confusing – clear” might correlate with the ratings of the SUS (see Fig. 9D). Therapist T3, who rated the system as complicated and confusing, explained that she would need more training to better understand the administering process and perceived the system instabilities that sometimes required rebooting as complicated.

The two aspects that the therapists liked most about the system were the integration of the Augmented Reality Gaming aspect, and the donning procedure of the robotic device. The magnetic coupling of the brace enables the therapists to don and adjust the brace in a comfortable position prior to connecting the arm to the robot, which was perceived as highly usable.

## Discussion

The objective was to evaluate the safety, clinical feasibility and usability, and potential benefits of a novel arm rehabilitation device based on an LBR robot and a serious game in AR (RobExReha system), from both a patient’s and therapist’s perspective. Key results indicate a safe use of the device with neurologically impaired patients and an acceptable to good usability reported by patients’ and therapists’ questionnaires. The combination of augmented reality using the HoloLens with a robotic arm training has shown to work for the patients that participated in the study. Furthermore, some features were identified that need improvement, such as an increase in system stability or better adjustability of the arm orthosis.

### Safety and clinical feasibility

The results suggest that the use of the RobExReha system was safe and feasible in neurological patients. None of the patients mentioned safety concerns. The occurrence of shoulder pain in one patient, however, highlights that special caution should be taken when treating patients with limited stability in the trunk and shoulder girdle with the RobexReha system.

The positioning of the LBR arm in the RobexReha system needs further adjustments to meet the needs of the patients’ range of motion, as it led to movement constrictions in some cases. The implementation of the compensation alert for detecting shoulder compensation proved to be useful once the threshold was properly adjusted. We believe that the alert successfully helped the patients to re-focus on the trunk stability without therapist intervention. Such functionality is very useful for enhancing self-efficacy and reducing the supervision effort.

The HoloLens was generally feasible and well-tolerated with only minor fitting issues.

### Donning and doffing

Most patients found that the device was relatively easy to put on. From a therapist’s perspective, only one therapist mentioned difficulties, specifically in the connection process of the elbow brace to the flange: sometimes the robot seemed to be in a slightly incorrect position. The large range of donning times of the HoloLens, from 10 to 151 s, reflects connection issues that were apparent in some cases. Despite this, the therapists valued the concept of the human-machine interface and perceived the donning and doffing times as acceptable. Particularly, the magnetic fixture of the elbow brace showed to be practicable in a clinical context, as it allows to don the brace in any comfortable position before attaching it to the robot. It also ensures a fast and easy disconnection from the robot. Regarding the donning and doffing times, this is supported by the results of Oliveira et al. [36] who reported an average of 206 s and 101 s for donning and doffing, respectively, for a robotic upper limb device; this was faster in donning and slower in doffing than the RobExReha procedure.

### Patients’ perspective

#### Usability questionnaires

Due to the prototype stage of the RobExReha device, we anticipated lower usability in the QUEST when compared to the Armeo Reference Group. However, this was only confirmed for the overall score and the items “reliability and safety” and “easy to use”. Yet, usability was generally rated positive by the patients in all questionnaires. Moreover, the mean QUEST score seems reasonable for a newly developed device, as it is comparable to the results from Guillén-Climent et al. who examined the usability of another novel rehabilitation device for upper extremity using robotics and serious gaming [37].

Features largely contributing to these lower ratings were the hand module (insufficient adjustability of length and wrist positions), the upper part of the arm orthosis (discomfort) and the HoloLens (too heavy or ill-fitting). The first two issues could be addressed during further developmental iterations of the robotic interface. Concerning the AR glasses, the HoloLens 2 (Microsoft Corp., US), might offer a greater field of view and improved comfortability. Additionally, the categories “reliability” and “safety” should be assessed separately in future evaluations, as lower perceived reliability largely contributed to the low ratings of this item, and no patient mentioned feeling unsafe at any moment.

#### Workload

The overall workload in the RTLX tended to be higher in the RobExReha group with a mean score of 41.7 compared to the Reference Group (mean: 34.1) without reaching a significant difference neither in the overall score nor domain-wise. Those are slightly lower compared to global work load scores, where video gaming had a median RTLX score of 56.5 [38], and to a serious gaming intervention in patients with chronic regional pain syndrome (mean 50.9) [39]. This could reflect that the demand of the robotic gaming therapy could potentially be higher for some patients. However, in neurorehabilitation the game design is particularly challenging, as it needs to be individually adaptable to both the patient’s physical and cognitive abilities. In this trial, due to the inclusion and exclusion criteria, the patients were fairly homogeneous in terms of mental abilities. Still, some patients needed some time and verbal explanation to understand the game. Therapeutic presence and guidance therefore were still vital aspects.

#### 3D perception

The gaming environment in AR was an important focus of this study. Specifically, concerns regarding the spatial perception of stroke patients when using the HoloLens were of interest. The feasibility of using the HoloLens in neurological patients as shown by Rohrbach et al. [28, 40], House et al. [15], Held et al. [16], Janssen et al. [17] and Höhler et al. [18] was confirmed and extended towards its application in combination with robotic support. Regarding the usability, we found the setup was generally practicable and the donning and doffing procedure fast. However, the donning procedure initially required a little practice, especially regarding the small field of view: if the HoloLens is not adjusted properly, it is likely that the patient can only see part of the projection area, limiting the field of view.

Regarding the AR perception by the patients, our results are very promising: All patients perceived or learned to perceive the holograms as three-dimensional and placed in space, with limited effort. Considering eight patients had impairments in stereoscopic vision, this is a remarkable finding. Still, the visual perception was challenging for one patient with reduced mobility of the cervical spine. As the field of view of the HoloLens is limited, the implemented game required the patients to explore the space in front of them actively by moving their head. In the implemented game, the holograms were placed in space, without linking them to physical objects within the room (e.g., placing a hologram on an actual table).

It can be hypothesized that the linking of the holograms onto real objects, preferably in a realistic setting (e.g., a table or shelf), could not only increase the meaningfulness of the game, but also facilitate the identification of the holograms in space. Nevertheless, as the individualized combination of different technologies guides the way to future rehabilitation [41], our results demonstrate the feasibility of combining robotics and AR-technology in patients with neurologically-induced upper limb deficits.

### Therapists’ perspective

The system was rated by the therapists in the UEQ-S as “leading edge”, “inventive”, “interesting” and “exciting”, despite some improvable features (as mentioned above) and the limitations in system stability. The overall usability as measured with the SUS was 60.5 ± 21.8 (25-82.5), which corresponds to an average of an “ok” to “good” usability [42]. This is slightly lower than the results of Pei et al. [43] who report an average SUS score of 71.8 (± 11.9) for their arm robot. Still, they also report quite a broad range in individual SUS scores, ranging from 42.5 to 92.5. This might indicate that the System Usability is highly affected by the respective performance of the device (i.e., did any errors occur during the respective sessions?) or the administrator’s skills and expectations (i.e., do I expect a device working perfectly? Am I technically skilled?).

Most likely, the downgrade in usability in this study arose from system stability issues (e.g., connection problems to the HoloLens, Robot Safety Stops) that sometimes required rebooting and thus made the administration of the device rather complex and time-consuming.

The RobExReha system provided a 2D-therapist view of the HoloLens view on a separate monitor. Still, most therapists remarked that they would have preferred to view the same holograms as the patient, to better support the patient.

### Potential optimization

Although the RobExReha system was generally perceived as inventive and usable, possibilities to improve the system could be identified:

1. The positioning of the LBR should be adjusted to better accommodate the targeted range of motion and avoid conflicts with the axis boundaries of the LBR arm.
2. Another solution for the weighing process for robotic calibration should be considered for the use in neurological patients, as pathological muscle tone deviations (e.g., spasticity) may be present. For example, an estimation derived from anthropometric tables combined with a feedforward model approach could resolve the problem [44].
3. For future scenarios, the chosen setup with the LBR arm would allow the inclusion of a resistance mode to enlarge the applicability. As such, patients with minor impairments to those with very limited arm function could use the device. The integration of a functional hand module would further add functionality to the robot and expand the gaming scenarios.
4. A second HoloLens for the supervising therapist would ease the support of the patient and opens possibilities of manipulating the holographic cues interactively.
5. Further gaming scenarios adapted to different levels of cognitive and motor function would enlarge the applicability of the system (e.g., simpler, and more complex gaming scenarios to motivate patients with different abilities).
6. The system stability should be increased to ensure good usability in clinical context.

### Limitations

Due to the prototype stage, the RobExReha system was only available for training of the right arm, which led to limitations in patient inclusion. The Reference Group, contrarily, additionally included patients with left-sided impairments. This could lead to bias; for example, in stroke patients, as the affected hemisphere influences the symptoms. Additionally, regarding the questionnaire evaluation, only patients with good cognitive abilities were enrolled in the study, which does not reflect the wide variation of neurological patients potentially training with robotic devices.

Another possible risk of bias arises as this study was not blinded and the patients, therapists and assessors were aware of which system they were evaluating. Due to shortages in personnel resources, sometimes a participant had to act as both an assessor and administering therapist, potentially leading to biased results in the questionnaires. However, given that this was a first proof-of-concept study, we consider the risk to be acceptable.

Further, regarding the study design as proof-of-concept study, no conclusions on efficacy of this type of therapy can be made.

## Conclusion

We investigated a novel robotic arm therapy system in combination with an augmented reality serious game. The system demonstrated to be feasible in patients with impairments of the upper extremity from neurological causes and good cognitive function. Notably, the good acceptance and perception of the game using the HoloLens is promising.

Despite the prototype stage of the RobExReha device, the system’s usability and safety was mostly rated not significantly different from an established device on the market, which is a considerable achievement. From a therapist’s perspective, the lack of reliability in terms of system instabilities largely contributed to lower usability ratings. Future developments of technology-assisted therapy devices in neurorehabilitation should thus focus on developing stable, simple, and easy-to-use systems.

## Data Availability

The dataset supporting the conclusion of this article is available on reasonable personal request.

## List of Abbreviations

AR: Augmented Reality
DRKS: Deutsches Register Klinischer Studien (German Clinical Trials Register)
LBR: Leichtbauroboter
RTLX: Raw Task Load Index
pAR: Presence in Augmented Reality Questionnaire
QUEST: Quebec User Evaluation of Satisfaction with assistive technology
ROM: Range of motion
SD: Standard deviation
SUS: System Usability Scale
UEQ: User Experience Questionnaire
